# An AIDS Patient With Pneumocystis jiroveci Pneumonia

**DOI:** 10.7759/cureus.86237

**Published:** 2025-06-17

**Authors:** Nicholas R Munoz, Armin Hojjat, Ajay Adial

**Affiliations:** 1 Internal Medicine, Temecula Valley Hospital, Temecula, USA; 2 Critical Care Medicine, Temecula Valley Hospital, Temecula, USA

**Keywords:** acquired immunodeficiency syndrome (aids), antiretroviral therapy, human immunodeficiency virus (hiv), infectious disease pathology, multifocal pneumonia, opportunistic fungal infection, pneumocystis jiroveci pneumonia

## Abstract

*Pneumocystis jiroveci* is a fungus that is a common opportunistic infection in immunocompromised patients, especially acquired immunodeficiency syndrome (AIDS) patients. First-line treatment is trimethoprim-sulfamethoxazole (TMP-SMX). We present the case of a 68-year-old male patient found to have AIDS after being non-compliant with human immunodeficiency virus (HIV) medications for 15 years. He had bilateral pneumonia that was found to be *Pneumocystis *pneumonia (PCP) caused by *Pneumocystis jiroveci*. He was treated with TMP-SMX and supportive therapy such as supplemental oxygenation and vasopressors. As his oxygen requirements decreased, he was weaned off vasopressors, his mentation improved, and he was subsequently downgraded from the ICU. PCP should be suspected in immunocompromised individuals with pneumonia. In AIDS patients with PCP, TMP-SMX is the treatment of choice. TMP-SMX should be continued prophylactically until CD4 counts improve.

## Introduction

*Pneumocystis jiroveci *(formerly known as *Pneumocystis carinii*) is a fungus associated with states of immunodeficiency; it is one of the most common infections associated with acquired immunodeficiency syndrome (AIDS). It often occurs in human immunodeficiency virus (HIV)-positive patients with low CD4 counts who are not receiving antiretroviral therapy or antimicrobial prophylaxis. Additionally, it is associated with organ transplantation, cancer, and medications that result in immune deficiency [1]. One study with 145 patients with *Pneumocystis *pneumonia (PCP) found that 79% had CD4 counts less than 100 cells/mm^3^ and 95% had CD4 counts less than 200 cells/mm^3^. Patients with CD4 counts below 200 cells/mm^3^ who are not on preventative therapy are nine times more likely to develop PCP than patients on prophylactic trimethoprim-sulfamethoxazole (TMP-SMX) [2]. Treatment for PCP is usually TMP-SMX; in some cases, steroids are indicated [3,4]. Herein, the case of a 68-year-old man with AIDS who was non-compliant with treatment and developed PCP is presented. He was diagnosed when his bronchoalveolar lavage (BAL) *Pneumocystis jiroveci* direct fluorescence antibody (DFA) smear returned positive. He was treated in the ICU with vasopressors and TMP-SMX. This case highlights the importance of considering PCP in immunocompromised patients, especially those with AIDS.

## Case presentation

**Figure 1:**
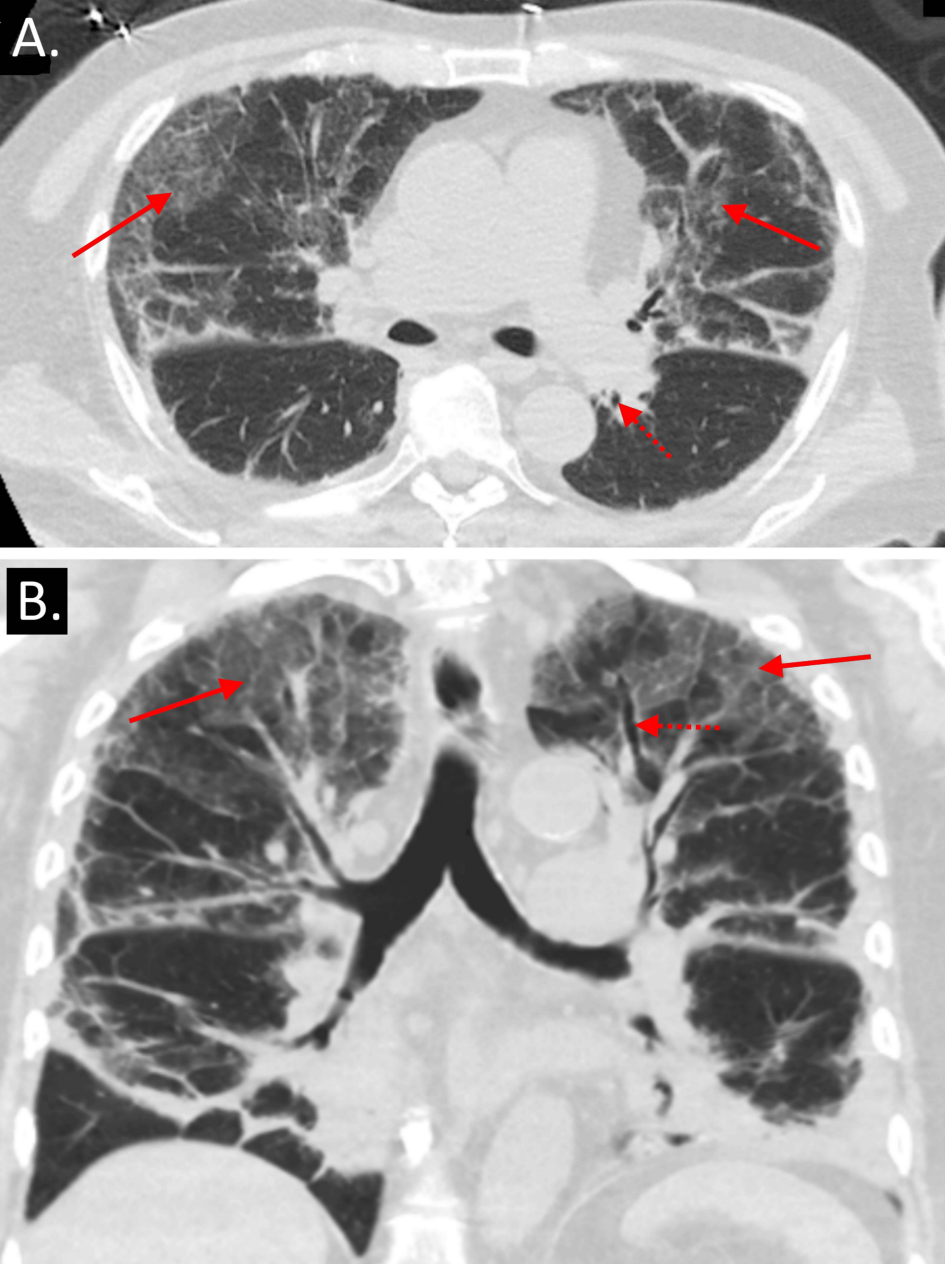
Transverse (A) and coronal (B) views of a non-contrast CT scan of the patient’s chest showing bilateral ground glass opacities (solid arrows) and bronchiectasis (dotted arrows)

The patient was a 68-year-old male with a history of HIV diagnosed 30 years prior, who had been non-compliant with HIV medications for 15 years. He originally presented to the emergency department with altered mental status, shortness of breath, and generalized weakness. He was found to be hypoxic and obtunded. Although he was arousable to tactile stimulation, he did not follow commands or answer questions. Labs were significant for leukocytosis of 14,000 cells/uL and a hemoglobin level of 13.1 g/dL (Table 1). Chest X-ray revealed patchy bilateral infiltrates and hyperinflated lungs. He was initially started on TMP-SMX, ceftriaxone, and dexamethasone. His respiratory status declined, and he went into septic shock. Subsequently, he was placed on 70% fraction of inspired oxygen (FiO_2_) at 15 L/min using a high-flow nasal cannula, started on a norepinephrine drip, and transferred to the ICU. His arterial oxygen pressure (PaO_2_) was found to be 61 mm Hg, prompting treatment with methylprednisolone. The following day, his hypotension improved, and he was weaned off norepinephrine. Follow-up CT scans of the thorax showed consolidation in the lung bases, scattered areas of ground-glass opacities, and bronchiectasis primarily in the upper lobes (Figure 1). Based on his history, he was presumed to have AIDS, and an infectious disease physician was consulted. To cover him for community-acquired pneumonia, atypical pneumonia, PCP, and cocci bacteria, he was started on broad-spectrum coverage with ceftriaxone, azithromycin, TMP-SMX, and fluconazole. After the initiation of antimicrobial therapy, he was found to be HIV positive with confirmed AIDS, as his CD4 count was 86 cells/mm^3^. To improve his respiratory status and diagnose the organism responsible for his pneumonia, fiber-optic bronchoscopy with BAL was performed. His BAL *Pneumocystis jiroveci *DFA smear was positive, and serum β-D-glucan was elevated at 218 pg/mL.

**Table 1:** The patient's laboratory values on admission (*) indicates an abnormal lab value. CO_2_: Carbon dioxide

Parameter	Patient's lab value	Reference range
White blood cells	14,000 cells/uL*	4,500 - 11,000 cells/uL
Hemoglobin	13.1 g/dL*	13.2 - 16.6 g/dL
Platelets	516,000 platelets/uL*	150,000 platelets/uL
Glucose level	111 mg/dL*	70 - 100 mg/dL
Sodium	142 mmol/L	135 - 145 mmol/L
Potassium	4.1 mmol/L	3.5 - 5.0 mmol/L
Chloride	105 mmol/L	96 - 106 mmol/L
CO_2_	25 mmol/L	21 - 32 mmol/L
Blood urea nitrogen	22 mg/dL	7 - 18 mg/dL
Creatinine	1.0 mg/dL	0.7 - 1.3 mg/dL

When the patient's *Pneumocystis jiroveci* DFA stain resulted positive, TMP-SMX at 5 mg/kg (of trimethoprim component) IV every six hours for 21 days was initiated, followed by TMP-SMX 800 mg/160 mg three times weekly for secondary prophylaxis. The ceftriaxone, azithromycin, and fluconazole were discontinued as his sputum cultures, blood cultures, and BAL specimens were negative for other organisms. With treatment, he was able to be weaned down to a 15 L/min oxymizer mask and was discharged from the ICU.

## Discussion

PCP is associated with states of immunodeficiency such as AIDS; the lower the CD4 count, the higher the incidence of the disease [2]. Clinical features include fever, cough, and dyspnea. CT scans have demonstrated high sensitivity for PCP in AIDS patients, and they may allow for the exclusion of PCP in individuals with normal findings [5]. While CT scans can assist in diagnosing PCP, a definitive diagnosis requires visualization of the organisms, as they cannot be cultured. The gold standard detection method for *Pneumocystis jiroveci* is DFA staining [6]. 

Treatment for PCP typically involves TMP-SMX, with severe infections necessitating IV medications, while mild to moderate infections can be treated orally. The standard dose of the drug is 15-20 mg/kg/day, administered in three or four divided doses [3]. Steroids are also linked to a reduction in mortality from PCP; a meta-analysis found a mortality rate of 15.2% in groups receiving steroids, compared to 27.7% in controls [2]. In patients with partial pressure of oxygen (PO_2_) < 70 mm Hg or an alveolar-arterial oxygen pressure (PAO_2_-PaO_2_) gradient ≥ 35 mm Hg, prednisone may be given at a dosage of 40-60 mg orally twice daily for five to seven days, followed by gradual reductions in the steroid [3,7]. 

In HIV patients, the most effective measure to prevent PCP is to improve CD4 counts through antiretroviral therapy (ART). Patients with a CD4 count < 200 cells/mm^3^ or a CD4 count < 14% should be considered for a prophylactic regimen of TMP-SMX. If a patient with HIV is not already on ART, it should be initiated within two weeks of a PCP diagnosis. The patient presented could likely have avoided PCP if he had been compliant with his ART. His best chance of avoiding reinfection would be to increase his CD4 count with ART and continue prophylactic TMP-SMX until his CD4 count rises [8,9]. The patient's presentation is consistent with infections in immunocompromised individuals and serves as a reminder to consider PCP in AIDS patients.

## Conclusions

The case of a patient who was non-compliant with his HIV medication, subsequently developed AIDS, and became infected with PCP is presented. He showed improvement after receiving TMP-SMX, with instructions to continue it until his CD4 count improves with appropriate ART to prevent reinfection with PCP and other opportunistic infections. In immunocompromised patients, particularly those with AIDS, *Pneumocystis jiroveci* infection should be considered. This case adds to a growing body of evidence regarding infections in immunocompromised patients.
